# Skeletal Muscle Contractile Function in Heart Failure With Reduced Ejection Fraction—A Focus on Nitric Oxide

**DOI:** 10.3389/fphys.2022.872719

**Published:** 2022-06-01

**Authors:** Lauren K. Park, Andrew R. Coggan, Linda R. Peterson

**Affiliations:** ^1^ Department of Medicine, Cardiology Division, Washington University School of Medicine, Saint Louis, MO, United States; ^2^ Department of Kinesiology, Indiana University Purdue University, Indianapolis, IN, United States

**Keywords:** heart failure, nitrate, nitrite, nitric oxide, skeletal muscle

## Abstract

Despite advances over the past few decades, heart failure with reduced ejection fraction (HFrEF) remains not only a mortal but a disabling disease. Indeed, the New York Heart Association classification of HFrEF severity is based on how much exercise a patient can perform. Moreover, exercise capacity—both aerobic exercise performance and muscle power—are intimately linked with survival in patients with HFrEF. This review will highlight the pathologic changes in skeletal muscle in HFrEF that are related to impaired exercise performance. Next, it will discuss the key role that impaired nitric oxide (NO) bioavailability plays in HFrEF skeletal muscle pathology. Lastly, it will discuss intriguing new data suggesting that the inorganic nitrate ‘enterosalivary pathway’ may be leveraged to increase NO bioavailability via ingestion of inorganic nitrate. This ingestion of inorganic nitrate has several advantages over organic nitrate (e.g., nitroglycerin) and the endogenous nitric oxide synthase pathway. Moreover, inorganic nitrate has been shown to improve exercise performance: both muscle power and aerobic capacity, in some recent small but well-controlled, cross-over studies in patients with HFrEF. Given the critical importance of better exercise performance for the amelioration of disability as well as its links with improved outcomes in patients with HFrEF, further studies of inorganic nitrate as a potential novel treatment is critical.

## 1 Introduction

Nearly six million men and women in the United States are affected by heart failure, a deadly disease whose impact is also significant across the globe (Mozaffarian et al., 2015). Approximately ½ of all such patients have *heart failure with reduced cardiac ejection fraction*, which will be the focus of this review and referred to as HF (Owan et al., 2006). Etiologies for HF can be grouped into myocardial injury (such as from coronary artery disease, myocarditis, toxins), abnormal loading conditions (e.g., hypertension, valvular heart disease), and arrhythmias (e.g., tachy- or bradyarrhythmias) (Murphy et al., 2020). A devastating characteristic of HF is its highly disabling effect that markedly impairs the ability of patients to perform normal activities of daily living (e.g., walking, standing from a sitting position, carrying groceries), which significantly reduces overall quality of life. Indeed, one of the major systems for characterizing the severity of HF, i.e., the New York Heart Association Functional Classification, categorizes HF based largely on patients’ ability to undertake physical activity, emphasizing diminished physical capacity as a primary characteristic of HF.

Historically, the decrease in exercise capacity accompanying a HF diagnosis was attributed to the inability of the failing heart to increase cardiac output sufficiently to provide adequate blood flow to the working muscles (Okita et al., 2013; Zizola and Schulze, 2013; Kennel et al., 2015). It is now abundantly clear, however, that secondary effects of HF on skeletal muscle morphological and contractile characteristics play a critical role in the diminished exercise capacity of patients with HF (Okita et al., 2013; Zizola and Schulze, 2013). In the present review, we discuss the evidence demonstrating alterations in intrinsic muscle contractile properties, i.e., in muscle strength, speed, and power, play an important role in limiting the functional capacity of patients with HF. We will then explore the mechanistic basis of muscle contractile dysfunction in HF, with specific attention being paid to the role of diminished nitric oxide (NO) bioavailability. Finally, we describe recent evidence indicating that increasing NO bioavailability via stimulation of the exogenous inorganic nitrate (NO_3_^-^) pathway by ingestion of NO_3_^-^ rich foods or supplementation may provide a simple but effective means of combating muscular dysfunction in HF.

## 2 Changes in Muscle and Its Contractile Function With HF

The earliest evidence that reduced exercise capacity in HF is not solely due to reduced cardiac function arose almost 40 years ago (Maskin et al., 1983; Wiener et al., 1986; Mancini et al., 1990). In particular, it was demonstrated that stimulation of β1 receptors via dobutamine infusion led to a positive ionotropic effect, increased cardiac output and limb blood flow but did not improve VO_2peak_ or exercise capacity in patients with HF (Maskin et al., 1983; Wiener et al., 1986; Mancini et al., 1990), nor did it correct abnormalities in muscle metabolism and function observed during exercise (Mancini et al., 1990). These observations led to studies using biopsies of the gastrocnemius and the vastus lateralis, which revealed marked structural and biochemical abnormalities in the muscles of patients with HF. Among these are atrophy of both type I, or slow-twitch, and type II, or fast-twitch, muscle fibers, along with an increase in the percentage of type II fibers and/or increased relative abundance of type II myosin (Mancini et al., 1989; Sullivan et al., 1990; Schaufelberger et al., 1995; Massie et al., 1996; Sullivan et al., 1997; Szentesi et al., 2005). These changes in fiber size and type are accompanied by a decline in capillarization (Sullivan et al., 1990; Schaufelberger et al., 1995), increases in glycolytic enzyme activity, as well as decreases in oxidative (mitochondrial) enzyme activity (Mancini et al., 1989; Sullivan et al., 1990; Szentesi et al., 2005). The latter is due to changes in both the quantity (total volume) and quality of mitochondria (Drexler et al., 1992). The net result is a diminished ability of muscle to generate ATP aerobically, leading to larger declines in phosphocreatine (PCr) and larger increases in inorganic phosphate (Pi) and H^+^ levels during exercise (Maskin et al., 1983; Wiener et al., 1986; Mancini et al., 1989). In turn, this leads to greater group III-IV afferent nerve signaling in patients with HF, thus accounting, at least in part and possibly even in full, for the abnormal ventilatory response and dyspnea during exercise characteristic of such patients (Olson et al., 2010; Keller-Ross et al., 2016). The exaggerated chemoreflex is thought to result in larger blood pressure responses to exercise in patients with HF, contributing to impaired exercise capacity (Kato et al., 2008). Indeed, increased feedback from group III-IV afferents even seems to constrain stroke volume during exercise in HF patients (Smith et al., 2020), thus potentially setting up a viscous cycle of reduced cardiac output → abnormal muscular metabolic responses → reduced cardiac output.

The muscular and hence metabolic/physiological abnormalities described above undoubtedly play an important role in the reduced exercise tolerance of patients with HF. A number of studies have demonstrated that, despite the increase in type II fiber percentage/myosin expression described above, the muscles of HF patients are weaker and slower, and hence less powerful, than those of healthy individuals (Buller et al., 1991; Magnusson et al., 1994; Harridge et al., 1996; Clark et al., 1997; Harrington et al., 1997; Sunnerhagen et al., 1998; Carrington et al., 2001; Brunjes et al., 2016). The plantar flexor muscles seem to be especially affected (Panizzolo et al., 2014; Panizzolo et al., 2015). This HF-related reduction in muscle contractility is apparent even when both groups are matched based on age, sex, physical activity, and medication (statin) use and differences in leg lean mass are incorporated into the statistical analysis (Toth et al., 2010), indicating that they are not merely due to inactivity and/or muscle atrophy (“cardiac cachexia”). Rather, they seem to be due in part to changes at the molecular level, which precede the decrease in whole-muscle function (Godard et al., 2012). In particular, studies using isolated single muscle fibers have demonstrated that HF results in a decrease in specific tension (i.e., force per cross-sectional area) and a reduction in maximal myosin ATPase activity (Szentesi et al., 2005). Other studies have shown a selective loss of myosin in type I, type IIA, and type IIA/X fibers (Miller et al., 2009) and a slowing of the rate of cross-bridge formation in both type I and IIA fibers and a reduction in Ca^2+^ sensitivity in IIA fibers (Miller et al., 2010). These abnormalities appear to be at least partially due to a decrease in Akt and/or mTOR phosphorylation (Toth et al., 2011). There are also HF-induced alterations in the content and/or regulation of Ca^2+^-handling proteins, i.e., ryanodine receptor type I, sarco(endo)plasmic reticulum Ca^2+^ ATPase (SERCA) 2a, phospholamban, and dihydropyridine receptor (Middlekauff et al., 2012; Rullman et al., 2013), which resemble those found in cardiac muscle (Andersson and Marks, 2010). Because contraction of skeletal muscle myocytes is critically dependent on cytoplasmic Ca^2+^, any alteration in the handling of Ca^2+^ would interfere with actin-myosin crossbridge formation, and therefore disrupt skeletal muscle force production. These molecular changes therefore help account for the reductions in whole-muscle contractile function found in patients with HF.

Thus, the altered muscle contractile properties of patients with HF have important implications for their quality and perhaps even length of life. This is because many normal activities of daily living (e.g., getting out of a chair, climbing a short flight of stairs) require generating significant power. HF also causes inspiratory (diaphragm) muscle weakness and fatigue that contributes to dyspnea and limited physical capacity (Empinado et al., 2014). Resistance exercise training has been found to significantly improve both objectively and subjectively measured physical function in HF patients (Savage et al., 2011). Muscle contractile function has also been demonstrated to be a better predictor of survival than VO_2_peak in HF patients (Hülsmann et al., 2004). Reductions in muscle contractility therefore play a key role in the morbidity and mortality of HF.

## 3 Reduced NO Bioavailability in HF and Impact on Exercise Capacity

Although numerous factors undoubtedly account for the decline in exercise performance and increased ventilatory effort in HFrEF, one key molecular factor contributing to these derangements is low NO bioavailability. This is evidenced by a significant reduction in breath NO levels in patients with HF (Adachi et al., 2003). Moreover, Katz et al., have demonstrated that HF results in reduced urinary excretion of [^15^N]nitrate after infusion of L-[^15^N]arginine, indicative of an overall decline in NOS activity (Katz et al., 1999). NO is also low in HF, at least in part, because of enhanced degradation by superoxide, the primary reactive oxygen species (ROS) created by the mitochondria, and potentially other ROS (Beckman and Koppenol, 1996; Münzel and Harrison, 1999; Cai and Harrison, 2000; Bertero and Maack, 2018). NO is well-known to contribute to vasodilation. Indeed, it was first called “endothelium-derived relaxing factor.” Thus, low NO bioavailability in HF likely contributes to the muscle’s impaired vasodilatory response to exercise and hence, to low tissue oxygenation and low V̇O_2peak_. However, NO is more than a vasodilator. NO has pleiotropic effects in many tissues, including skeletal muscle. In muscle, NO helps modulate contractile function (cf ((Maréchal and Gailly, 1999; Stamler and Meissner, 2001; Suhr et al., 2013) for review). Directly, NO may slightly suppress isometric force production due to S-nitrosation of specific proteins (Lamb and Westerblad, 2011). However, the following indirect effects of NO overcome this possible suppression, resulting in enhanced skeletal muscle function. NO increases the rate of force development, maximal shortening velocity, and maximal power of single muscle fibers and isolated muscle (Maréchal and Gailly, 1999; Kaminski and Andrade, 2001; Stamler and Meissner, 2001; Suhr et al., 2013). NO apparently enhances skeletal muscle function via increasing soluble guanylate cyclase (sGC) activity and hence cyclic guanosine monophosphate (cGMP) production, effectively reversing the abnormalities previously described (Maréchal and Gailly, 1999). Thus, increasing the low NO levels in HFrEF patients should improve peripheral vascular and skeletal muscle function, resulting in improved exercise performance and reduced ventilatory effort.

## 4 Nitric Oxide Generation Pathways With a Focus on the Exogenous NO_3_^-^ Pathway

NO was first discovered by Joseph Priestly in 1772. However, for much of the time since its discovery NO has been thought of as merely a pollutant because it destroys ozone and is a component of acid rain and smog. Finally, in the 1980s, researchers interested in blood flow discovered the important role that NO plays in vasodilation and human health. There are three main pathways for the delivery of NO to skeletal muscle: the endogenous (nitric oxide synthase) pathway, the exogenous organic nitrate pathway, and the exogenous inorganic enterosalivary pathway. The endogenous pathway was first described by Furchgott, Ignarro, and Murad for which they received the 1998 Nobel Prize in Physiology or Medicine. This Nobel prize-winning discovery elucidated that the previously described “endothelium relaxing factor” was NO, which was generated by a family of enzymes known as nitric oxide synthases (NOS). The primary substrate for NOS is the semi-essential amino acid L-arginine. NOS catalyzes the following reaction to create NO: 2 L-arginine + 3 NADPH +3 H^+^ + 4 O_2_ ⇌ two citrulline +2 NO + 4 H_2_O+ 3 NADP^+^. There are several mammalian NOS isoenzymes, i.e., NOS1 or neuronal NOS (nNOS), NOS2 or inducible NOS (iNOS), and NOS3 or endothelial NOS (eNOS). A detailed description of the different NOS subtypes and their locations and function is beyond the scope of this review but have been recently discussed in detail by Król and Kepinska (Król and Kepinska, 2020).

There are several known benefits to organic nitrates in HF. First, they are venodilators, lowering venous pressure and thereby minimizing edema due to extravasation of water from the vascular system. Second, organic nitrates can also cause arterial vasodilation, thereby decreasing afterload on the failing heart and improving coronary blood flow. Improved epicardial coronary blood flow is especially beneficial in the setting of ischemic cardiomyopathy. Improved coronary blood flow may lead to an improvement in myocardial tissue perfusion, which is associated with improved outcomes in patients with HF, even in the absence of epicardial coronary artery disease (Kadkhodayan et al., 2017). The benefits of organic nitrates in patients with HF are most pronounced in those who are Black, as they demonstrated an improvement in outcomes, including overall survival, when organic nitrates were paired with the afterload reducing agent hydralazine in the V-HeFT and A-HeFT trials (Taylor et al., 2004; Echols and Yancy, 2006). Because of the results from these trials, the combination is hydralazine and isosorbide dinitrate are a class 1 indication for patients with HF who self-identify as Black or African American. In other patients with HF, who cannot be given first-line HF agents, the combination of isosorbide dinitrate + hydralazine has a class 2b indication (Aguilar et al., 2022). There are several limitations to the use of organic nitrates, however. Hypotension, headache, and the development of ‘tolerance’ can all limit the use of organic nitrates in patients (Münzel et al., 2014). Also, to our knowledge there are no studies to date that have evaluated the effect of organic nitrates specifically on skeletal muscle contractile function in patients with HF.

**FIGURE 1:**
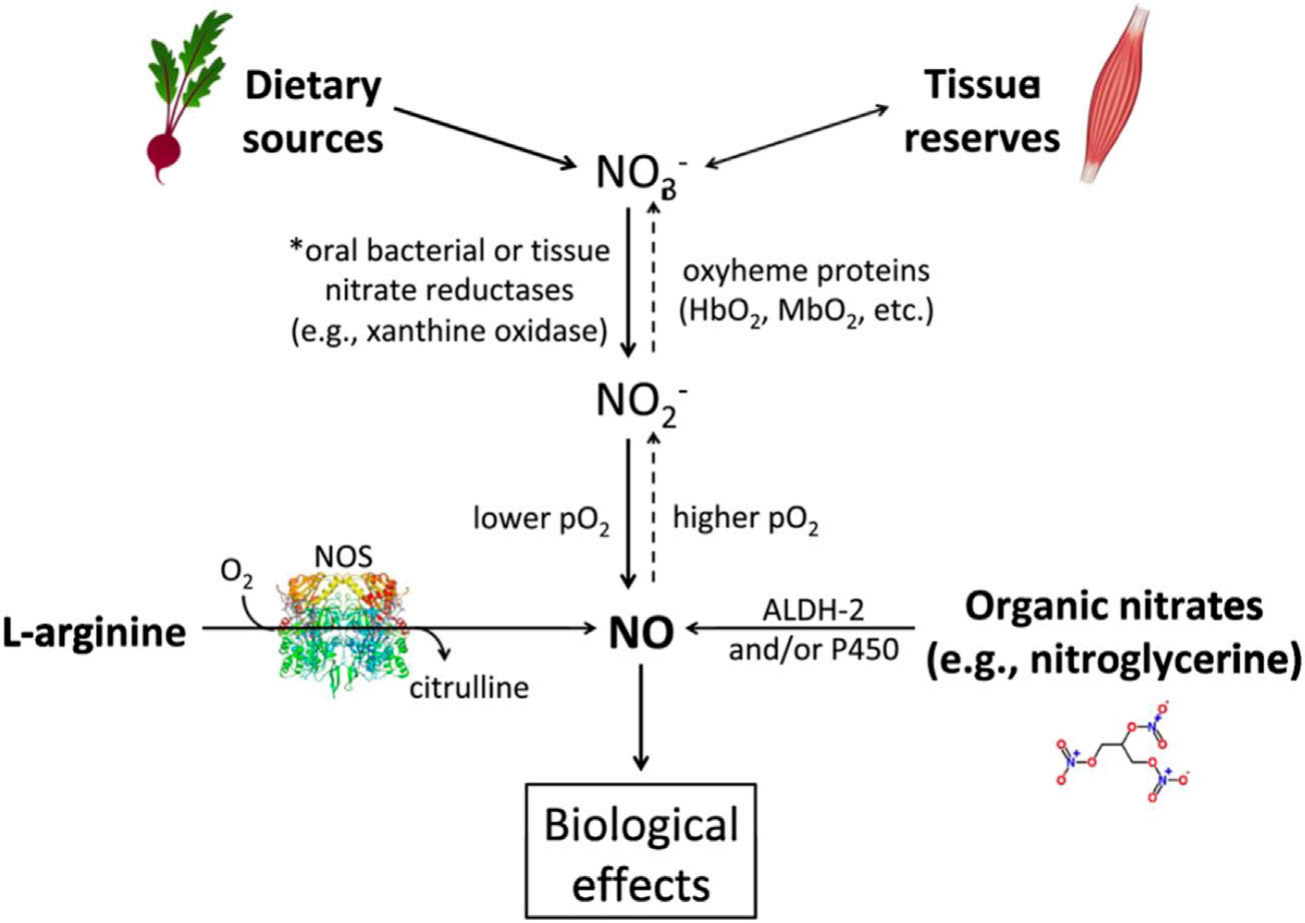
The main pathways for nitric oxide (NO) production. The dietary pathway (starting at upper left) utilizes NO_3_^-^ and is facilitated by lower pO_2_ and pH. Ingestion of NO_3_^−^-containing foods, especially vegetables such as beetroot, start this pathway. NO_3_^−^ is reduced to nitrite (NO_2_^-^) by reductases or acidic conditions and facilitated by oxyheme proteins. Then NO_2_^-^ is reduced to NO under the appropriate conditions. Importantly, skeletal muscle can serve as a ‘reservoir’ for nitrate. As shown by the dashed arrows, this pathway can also ‘run in reverse’ with NO being used to create NO_2_^-^ and then NO_3_^−^ given the appropriate conditions. *Note: NO_3_^−^ can be taken up from the circulation into the salivary glands and go through this reduction pathway again in what is known as ‘the enterosalivary pathway.’ The endogenous pathway (lower left) uses NO synthase and oxygen to create citrulline and NO. An abbreviated depiction of the organic nitrate pathway (lower right) shows the production of NO derived from pharmacologic sources, such as nitroglycerin. ALDH-2 = aldehyde dehydrogenase, P450 = cytochrome P450. Reproduced with permission from Biochimica et Biophysica Acta (BBA)—Molecular Basis of Disease. Vol 1865, Mulkareddy V, Racette SB, Coggan AR, Peterson LR. Dietary nitrate’s effects on exercise performance in heart failure with reduced ejection fraction (HF). Page Nos 735–740. Copyright Elsevier (2019).

**FIGURE 2:**
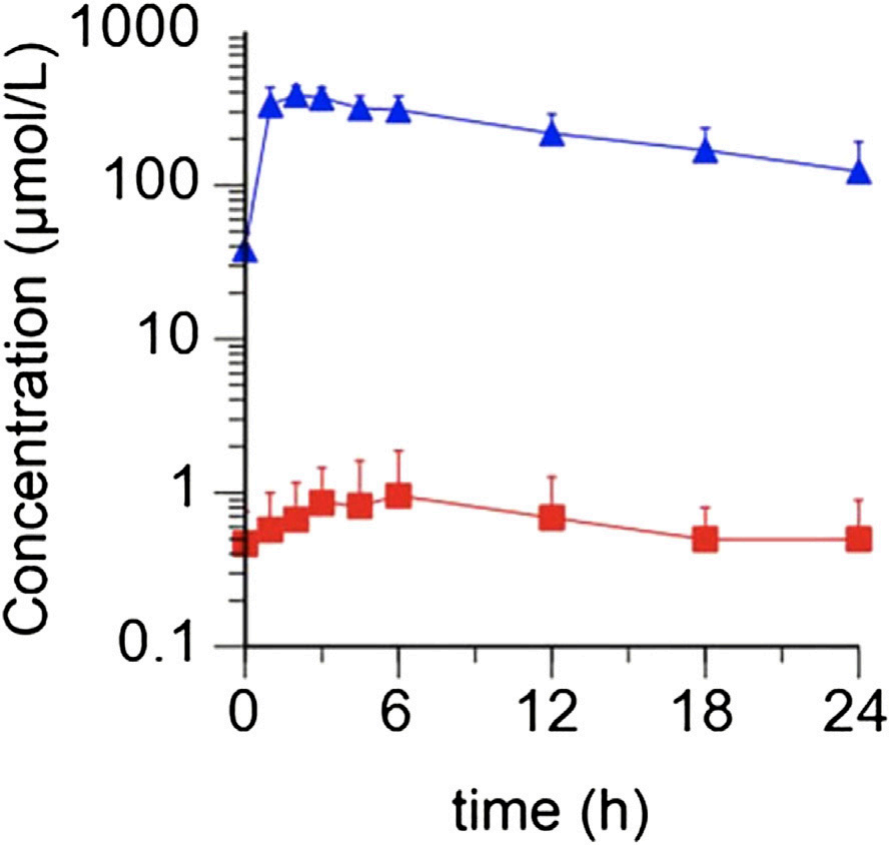
Average concentrations of plasma NO_3_^-^ and NO_2_^-^ observed in patients (*N* = 5) following a 10 mmol dose of KNO_3_. NO_3_^-^ concentrations are depicted as blue triangles, and NO_2_^-^ concentrations as red squares (Reproduced with permission from *Pharmacology Research*).

In contrast, there is a burgeoning literature on the effects of exogenous (inorganic) NO_3_^-^ on skeletal muscle function, including in patients with HF. The discovery of exogenous NO_3_^-^ as a source of NO for the skeletal muscle and other organs was far more recent than the other two pathways for NO production. It was only in 1994 that the ‘enterosalivary pathway for the production of NO from NO_3_^-^ was recognized. NO_3_^-^ is found in drinking water, especially from contaminated wells, which was previously thought to be associated with increased risk of cancer, though this association has long been disproved (L'Hirondel et al., 2006). In fact, most NO_3_^-^ is obtained from food (see Figure 1), particularly beetroot and dark green leafy vegetables. After ingestion, NO_3_^-^ enters the “enterosalivary pathway” (Kapil et al., 2010). In the mouth (and the rest of the gut) facultative anaerobes, such as *Veilonella*, help reduce NO_3_^-^ to nitrite (NO_2_^-^) (Kapil et al., 2010). Both NO_3_^-^ and NO_2_^-^ are swallowed and enter the stomach. The stomach’s acidic conditions facilitate reduction of NO_3_^-^ and NO_2_^-^ and NO_2_^-^ to NO (In fact, proton pump inhibitors abolish some of the physiological effects of NO_3_^-^) (Montenegro et al., 2017). NO_3_^-^ and NO_2_^-^ are then absorbed by the gastrointestinal tract and enter the circulation and hence, other tissues. Skeletal muscle in particular is thought to be a major ‘sink’ for NO_3_^-^ (Mulkareddy et al., 2019). The NO_3_^-^ to NO_2_^-^ to NO conversion in the skeletal muscle is facilitated by low pH and anaerobic conditions, which are often seen in heavily exercising skeletal muscle (Piknova et al., 2015). The molecule NO has pleiotropic effects, but of particular importance to skeletal muscle and the smooth muscle surrounding arteries, NO stimulates the activation of soluble guanylate cyclase. This increases cyclic guanyl monophosphate (cGMP), which activates several phosphodiesterases, ion channels, and kinases (Kapil et al., 2010). This chain reaction is well-known to lead to smooth muscle *relaxation*, and consequent vasodilation. It is less clear how NO enhances skeletal muscle contractile function, but it appears to be due to alterations in calcium release and/or sensitivity, possibly through enhanced activation of cGMP (Coggan and Peterson, 2018). NO is thought to be short-lived and can also be oxidized and converted back to NO_2_^-^ and to NO_3_^-^. This results in recycling of NO_3_^-^, allowing for prolonged exposure of tissues to NO_3_^-^ and its reduction products—NO_2_^-^ and NO. Preliminary data from patients with HF show that the time to peak plasma NO_3_^-^ concentrations is ∼1–3 h after ingestion with a slow taper over 24 h. Plasma NO_2_^-^ concentrations peak slightly later, i.e., ∼3–6 h after ingestion (see Figure 2). This mirrors the time course of plasma NO_3_^-^ and NO_2_^-^ described in the literature in patients with hypertension (Kapil et al., 2010). Based on these pharmacokinetic data most studies of NO_3_^-^ effects on skeletal muscle and exercise performance have been performed ∼2–3 h after ingestion with NO_3_^-^.

## 5 Effect of NO_3_^-^ on Exercise Performance in HF

The acute effects on skeletal muscle power in the short time after ingestion in patients with HF are significant and are quantitatively greater than those of healthy individuals. In a double-blind, randomized crossover study of healthy subjects, knee extensor average muscle power and muscle contraction Percent increase in Vmax was 11% after a single dose of 11.2 mmol of NO_3_^-^ (Coggan et al., 2015a). Similarly, a study of athletes showed maximum power (assessed using a maximal inertial-load cycling trial) improved ∼6% after a single dose of NO_3_^-^ supplementation (Rimer et al., 2016). Patients with HF, though, appeared to derive an even greater benefit from an acute dose of NO_3_^-^. In a double-blind, placebo-controlled, crossover study of patients with HF, knee extensor power was increased by 11% at the highest velocity tested (6.28 rad/s) and trended toward a 9% improvement at 4.71 rad/s (Coggan et al., 2015b). Interestingly, this more marked improvement in muscle power in patients with HF, as compared with healthy subjects, was evident despite the patients having both a lower baseline breath NO and less of an increase in breath NO after ingestion of NO_3_^-^. This lower baseline breath NO and lesser rise in breath NO may be due to greater destruction of NO from oxidative stress and/or from differences in nitrate reduction or absorption (Coggan et al., 2015b). This indirectly supports the hypothesis that impaired muscle power in HF may be partly due to decreases in NO bioavailability (Coggan et al., 2015b). The improvements in muscle power in patients with HF are not the only improvements in physical performance associated with NO_3_^-^, however.

There are also data to support the idea that NO_3_^-^ supplementation can improve aerobic exercise performance in patients with HF. Studies of aerobic performance in patients was based on earlier studies in healthy subjects, which have often (but not always) shown improvements in exercise efficiency, possibly due to direct inhibition of mitochondrial respiration or a decrease in ATP utilization (Bailey et al., 2010; Larsen et al., 2011; Coggan and Peterson, 2018). However, in a double-blind, placebo-controlled, crossover study of NO_3_^-^ (11.2 mmol in the form of beetroot juice) in patients with HF (mean left ventricular ejection fraction 34±2%), our group showed that exercise efficiency (the ratio of external power to metabolic power calculated utilizing respiratory gas exchange data collected during a submaximal exercise test) was not changed with an acute dose of 11.2 mmol NO_3_^-^ treatment (Coggan et al., 2018). Nevertheless, in this study VO_2_peak and maximal cycling exercise duration significantly increased (Coggan et al., 2018); this was mirrored by an increase in plasma NO_3_^-^ and NO_2_^-^ (Coggan et al., 2018). Though the increase in VO_2_peak (∼1.6±0.5 mL min^−1^kg^−1^) was relatively modest, this would correspond to a clinically significant change because studies suggest that for every increase of 1 mL min^-1^ kg^−1^ there is a 5% decrease in the annualized risk of transplantation/death (Peterson et al., 2003). The improvement seen in VO_2_peak is congruent with the findings of Kerley et al., who found an improvement in exercise performance as measured by an incremental shuttle test in patients with nonischemic HF (Kerley et al., 2016). However, in a study by Hirai et al., repeated intake of NO_3_^-^ did not result in an improvement in VO_2_peak in patients with predominantly ischemic cardiomyopathy (Hirai et al., 2017). The reason(s) for the discrepancy in findings among these studies is not clear, but it may be that disease etiology plays a role in responsiveness to NO_3_^-^ (Coggan et al., 2018).

Importantly, these improvements in VO_2_peak, total exercise time, and skeletal muscle power have all been in addition to that which would be seen from guideline-directed medical therapy (GDMT). As shown in Table 1, the improvements in VO_2_peak seen with an *acute* dose of NO_3_^-^ therapy are just slightly less than the improvements that would be seen from *chronic* beta-blocker therapy or from *chronic* angiotensin converting enzyme (ACE)-inhibitor or angiotensin receptor blocker (ARB) therapy and more that what is expected from *chronic* aldosterone antagonism. Moreover, the muscle power improvements seen after ingestion of acute dietary nitrate are in stark contrast to no change in muscle power after *chronic* ACE-inhibitor/ARB or beta-blocker therapy. It is also important to note as well that in several small trials inorganic nitrate therapy has not resulted in hypotension in subjects already taking ACE-inhibitor/ARB and/or beta-blocker therapy (Coggan et al., 2015b; Coggan et al., 2018; Coggan et al., 2020).

**TABLE 1:** Different pathways for enhancing NO and advantages of KNO_3_.

Alternative Approach	Advantages of KNO_3_
**L-arginine**	Not dependent on NO synthase
	Functions well in acidic tissue
	Functions well in ischemic tissue
**Organic, pharmacologic nitrates e.g., nitroglycerin**	Does not cause tolerance
	Does not increase reactive oxygen species (ROS) in mitochondria
	May be less likely to cause hypotension
	May be less likely to cause headaches
**Sildenafil or other phosphodiesterase inhibitors**	Inadvertent inhibition of PDE6 (Phospodiesterase 6), which is thought to be responsible for retinal dysfunction and vision changes
	May be less likely to cause hypotension
	May be less likely to cause flushing or headache
**Beetroot Juice**	Does not contain oxalate (decreased risk of kidney stones)
	No allergies
	No bitter taste
	Easier to control exact NO_3_^-^ content
	More portable
	No beeturia to be confused with/mask renal/urinary tract disease
	No red stool to be confused with/mask gastrointestinal disease

## 6 Form, Dose, and Timing of NO_3_^-^ Supplementation

Many of the studies of NO_3_^-^ efficacy on exercise performance were done using concentrated beetroot juice as the source of nitrate, but there are other formulations that have been studied. An advantage of using beetroot juice is that it is a natural foodstuff, which has increased appeal to a segment of the population and does not require a prescription from a physician. There are several different formulations of beetroot juice, as well as beet powder (with added nitrite) and other food sources of NO_3_^-^ (Gallardo and Coggan, 2019). Concentrated beetroot juices have an advantage over nonconcentrated juices for the treatment of HF because less volume needs to be consumed to get the requisite amount of NO_3_^-^. For research purposes, the commercial brand ‘Beet It’ concentrated beetroot juice makes an identical-appearing placebo that is devoid of NO_3_^-^, which facilitates conduct of true double-blind, placebo-controlled trials. There are some disadvantages of using beetroot juice or other foodstuff as the vehicle for delivering NO_3_^-^, however. First, there is a wide range of NO_3_^-^ amongst various brands (Gallardo and Coggan, 2019). There is also potentially more variation within brands than there would be in a regulated prescription pill that is FDA-approved. There are also a few other potential disadvantages to using beetroot juice as a source for NO_3_^-^ delivery to patients. Beeturia (red coloration of urine) and red stools are common after ingestion of beets. This can not only potentially cause concern for patients who may think they are bleeding but also could mask if they were actually bleeding. While not a major problem for an acute dose of beetroot juice, this could be a problem if this were prescribed as a treatment to be taken daily for HF. Beets are also a source of oxalate, which could theoretically possibly increase the risk of kidney stones, especially if taken daily for years as a treatment for HF. Lastly, some patients may not like the taste of beets, and even concentrated beetroot juice bottles are larger and potentially less portable than a pill format. Thus, some studies of patients with hypertension and patients with HF have moved to using nitrate salts (cation + NO_3_) in a pill format; e.g., KNO_3_ pills to obviate some of the disadvantages of beetroot juice (Table 2) (Coggan et al., 2020; Sundqvist et al., 2020). KNO_3_ (aka saltpeter or niter) has been known to humans since prehistoric times and has been used in Chinese medicine since the 8^th^ century for treatment of angina (Butler and Feelisch, 2008). Others have used NaNO_3_ supplementation, intravenous infusions or inhaled NO_2_^-^ in healthy volunteers or those with hypertension or heart failure with preserved ejection fraction (Larsen et al., 2006; Borlaug et al., 2015; Bashline et al., 2020). To our knowledge, no formulations other than beetroot juice or KNO_3_ have been used in studies of HF. A disadvantage of infusions or inhaled NO_2_^-^ is potential cost or cumbersome apparatus for administration for outpatients. Another disadvantage of administration of NO_2_^-^ is its very short half-life (i.e., ∼30 min) (Dejam et al., 2007; Hunault et al., 2009), which would requiring impractically frequent dosing to maintain plasma levels. This limitation can be partially overcome by administering greater amounts of NO_2_^-^, but this results in headache, nausea, and/or methemoglobinemia in a significant percentage of individuals (Justice et al., 2015). In sum, there are many different routes and forms of NO_3_^-^/NO_2_^-^ that can be potentially leveraged to boost NO production via the exogenous pathway for HF.

**TABLE 2:** Improvements in VO_2_peak and muscle power after dietary NO_3_^-^ vs common classes of HF drugs in patients with HF with reduced ejection fraction.

	*Acute Inorganic NO_3_^-^ Intake	Chronic beta-Adrenergic Blockade	*Chronic* ACEI/ARB Use	*Chronic* Aldosterone Antagonism
VO_2_peak	6%	9%	10%	0
Muscle Power	9–13%	N/c	N/c	N/a

a that the benefits of *acute* NO_3_^-^ are in addition to *chronic* beta-blocker use + Angiotensin Converting enzyme-inhibitor (ACEI)/Angiotensin receptor blocker (ARB) and aldosterone antagonists. N/c = no change; N/a = data not available.

## 7 Conclusions and Clinical Relevance

In HF the underlying problem starts off as a single-organ problem: that of reduced cardiac function and output. However, the cascade of the neurohumoral activation and downstream effects of decreased cardiac output affect many organs including skeletal muscle. This multi-organ dysfunction then feeds-forward on the pathophysiology and symptomatology of HF. For example, derangements of skeletal muscle function exacerbate the impaired physical functioning of patients with HF. Skeletal muscle structure and signaling are both impaired in HF, and a primary signaling derangement is a deficiency of NO. Endogenously derived NO appears to be diminished due to decreased production and increased destruction. While organic nitrates, such as nitroglycerin have been used for over a century, they have limitations—including the development of tolerance with prolonged continuous use. Recently, NO_3_^-^ (ingested and circulated via the enterosalivary pathway) has been leveraged to increase NO bioavailability and skeletal muscle performance. NO is well-known for increasing perfusion by inducing *relaxation* of smooth muscle (around arteries), but NO also *stimulates* skeletal muscle. Different formulations and delivery routes of NO_3_^-^ (as well as nitrite) have been used in studies of HF, hypertension, and heart failure with preserved ejection fraction. The advantages and disadvantages of these different modes/formulas has been described above. There are now several small proof-of-concept studies showing intriguing improvements in physical performance—both muscle power and aerobic performance—in patients with HF after an acute dose of NO_3_^-^. Future, larger trials are needed for further proof that NO_3_^-^ may restore NO bioavailability thereby improving physical performance and disability from HF have been used for over a century, they have limitations—including the development of tolerance with prolonged continuous use. Recently, NO_3_^-^ (ingested and circulated via the enterosalivary pathway) has been leveraged to increase NO bioavailability and skeletal muscle performance. NO is well-known for increasing perfusion by inducing *relaxation* of smooth muscle (around arteries), but NO also *stimulates* skeletal muscle.
